# CD123 and More: How to Target the Cell Surface of Blastic Plasmacytoid Dendritic Cell Neoplasm

**DOI:** 10.3390/cancers14092287

**Published:** 2022-05-03

**Authors:** Elodie Bôle-Richard, Naveen Pemmaraju, Blandine Caël, Etienne Daguindau, Andrew A. Lane

**Affiliations:** 1 INSERM, EFS BFC, UMR1098, RIGHT, University of Bourgogne Franche-Comté, Interactions Greffon-Hôte-Tumeur/Ingénierie Cellulaire et Génique, F-25000 Besancon, France; blandine.cael.ext@efs.sante.fr (B.C.); edaguindau@chu-besancon.fr (E.D.); 2 Department of Leukemia, University of Texas MD Anderson Cancer Center, Houston, TX 77030, USA; npemmaraju@mdanderson.org; 3 Service Hématologie, CHU Besançon, F-25000 Besancon, France; 4 Department of Medical Oncology, Dana-Farber Cancer Institute, Harvard Medical School, Boston, MA 02215, USA

**Keywords:** BPDCN, leukemia, AML, CD123, tagraxofusp, bispecific antibody, CAR-T cell

## Abstract

**Simple Summary:**

Until recently, there were no approved therapies for the aggressive blood cancer blastic plasmacytoid dendritic cell neoplasm (BPDCN). Survival for patients diagnosed with BPDCN is under two years, and improved treatments are needed. In 2018, tagraxofusp became the first approved drug for BPDCN. Tagraxofusp is an interleukin 3-dipththeria toxin recombinant fusion protein that targets CD123, a component of the interleukin 3 receptor, on the surface of BPDCN cells. Here, we discuss the development of tagraxofusp and other newer agents that also target CD123. We also present rationale for several other cell surface proteins, expressed on BPDCN, that are targets for therapies already in development for other cancers and that might be also considered for evaluation in BPDCN.

**Abstract:**

Blastic plasmacytoid dendritic cell neoplasm (BPDCN) is a rare and aggressive leukemia derived from plasmacytoid dendritic cells (pDCs). It is associated with a remarkably poor prognosis and unmet need for better therapies. Recently, the first-in-class CD123-targeting therapy, tagraxofusp, was approved for treatment of BPDCN. Other CD123-targeting strategies are in development, including bispecific antibodies and combination approaches with tagraxofusp and other novel agents. In other blood cancers, adoptive T-cell therapy using chimeric antigen receptor (CAR)-modified T cells represents a promising new avenue in immunotherapy, showing durable remissions in some relapsed hematologic malignancies. Here, we report on novel and innovative therapies in development to target surface molecules in BPDCN currently in clinical trials or in preclinical stages. We also discuss new cell surface targets that may have implications for future BPDCN treatment.

## 1. Introduction

Blastic plasmacytoid dendritic cell neoplasm (BPDCN) is a rare and aggressive leukemia characterized by a clonal expansion of plasmacytoid dendritic cells (pDCs). There has not historically been a standard-of-care therapy for these patients, and they were treated with leukemia or lymphoma chemotherapies. However, conventional chemotherapy is largely inadequate in BPDCN, since although many patients initially respond, the responses are short-lived and relapsed BPDCN is quite chemoresistant. This has prompted the evaluation of alternative therapies such as targeted cytotoxins and immunotherapy. Recently, tagraxofusp became the first drug approved for BPDCN in the US and Europe. Tagraxofusp is an interleukin 3-diphtheria toxin fusion protein that targets the IL3 receptor alpha subunit, or CD123, which is highly expressed on the surface of BPDCN cells [1]. Some patients do not tolerate tagraxofusp or it may not be available, and therefore standard chemotherapy is still used. The most efficient regimens are broadly separated into these sub-types: acute leukemia treatment (AML or ALL-like), lymphoma-like treatment, and asparaginase/methotrexate-based treatments that are also used in aggressive leukemias and lymphomas. Retrospective data imply a better rate of response with first-line therapy that includes asparaginase/MTX and in ALL-like compared to AML-like or lymphoma-like treatment. However, this assumption would ideally be confirmed in prospective cohorts. Beyond those “classical” chemotherapies, innovative approaches have been developed through the integration of data on the physiopathology and oncogenesis of BPDCN. Proof of concept of the efficacy of targeted therapies such as bortezomib or venetoclax has been recently provided with in vitro models and pre-clinical data [2,3,4,5]. The recent increased use of BCL-2 inhibition in myeloid malignancies and its approval in acute myeloid leukemia along with azacitidine lead to the assessment of the venetoclax alone or in combination in ongoing clinical trials enrolling patients with BPDCN.

Even if most patients respond to initial tagraxofusp or chemotherapy, relapse is common without consolidation by hematopoietic cell transplantation (HCT). In the setting of HCT, overall survival can reach 40% after a follow-up of 5 years [6,7,8,9,10]. However, relapse still occurs in at least 2/3 of allograft patients. Transplantation in first complete remission (CR1) is associated with a better outcome compared to patients not in CR1. However, HCT is associated with a significative toxicity that limits its indication to fit and younger patients. Altogether, the data reporting outcomes with chemotherapy and HCT demonstrate a large medical need for refractory patients and/or elderly patients that may not be able to undergo intensive treatments. The long-lasting remissions that have been obtained after HCT demonstrate the curative potential of adoptive therapies or immunotherapies and support the design of such novel approaches.

Anti-tumor immunotherapy is a therapeutic strategy based on the principle of immunosurveillance of cancers: since the immune system is naturally capable of recognizing cancer cells and destroying them, immunotherapy consists of artificially mobilizing immune cells to recognize and eliminate malignant cells [11]. Thanks to the various clinical successes that have occurred, immunotherapy has been considered a major breakthrough in terms of cancer treatment [12]. Today, there are different types of immunotherapy: passive immunotherapy based on monoclonal antibodies, active immunotherapy using cytokines such as IFNα, IL-2, or TNF, and adoptive immunotherapy that uses effectors of the immune response such as T cells. Indeed, over the last twenty-five years, new cellular therapies against cancer based on the ex vivo manipulation and re-infusion of autologous or allogeneic immune cells have been widely tested in the clinic. These different immunotherapy approaches have shown great potential for the treatment of cancers, especially those resistant to conventional therapies (surgery, radiotherapy, and chemotherapy) [13].

## 2. Targeted Therapies with Results or in Evaluation for BPDCN

### 2.1. Tagraxofusp

CD123, the interleukin (IL)-3 receptor alpha chain, is overexpressed in 100% of BPDCN cases, and not expressed or weakly expressed on normal cells other than normal plasmacytoid dendritic cells and basophils [14,15]. CD123 was identified as a promising therapeutic target for BPDCN patients. Frankel et al. launched the first pilot studies investigating a CD123-targeted agent which featured a novel recombinant protein drug consisting of a modified diphtheria toxin payload that was fused to recombinant human IL-3 [16]. The unique drug construct, originally known as “DT-IL3”, was tested initially in patients with MDS and AML, and the trial also included a small number of patients with BPDCN [17]. Although the drug was found to have modest single-agent activity in AML and MDS in these early clinical trials, what stood out was the early efficacy signal in BPDCN [18]. Therefore, Frankel et al. embarked on a pilot early phase study specifically focusing on patients with BPDCN with the same targeted therapy, which was renamed SL-401 (Stemline Therapeutics, New York, NY, USA) [19]. In this clinical trial, a total of 11 patients, all male with a median age of ~70 years, were enrolled. Most patients were only able to receive one cycle (5 doses, days 1–5) of therapy. Among the 11 patients, 9 were deemed eligible for evaluation. The authors reported an overall response rate (ORR) of 78% (7 of 9 patients responding), including five complete responses (CRs) and two partial responses (PRs). Toxicity was notable for the vascular leak syndrome (VLS), which is now known as the capillary leak syndrome (CLS). The authors noted that CLS was found to be manageable overall and tracked with low or decreased serum albumin and weight gain [19]. The accompanying editorial by Fitzgerald noted the novel agent as a potential breakthrough in the rare disease field of BPDCN [18].

Building upon the momentum generated by these early results, Pemmaraju et al. sought to further investigate this agent in a larger population of patients with BPDCN [1]. They conducted the first prospective, multi-institutional study of a targeted agent in BPDCN using this agent, SL-401 (now known as tagraxofusp). Including both frontline (FL) and relapsed/refractory (R/R) subjects, this four-stage study consisted of: (1) a dose-escalation 3 + 3 design (FL and R/R) for safety; (2) an expansion stage (FL and R/R); (3) a pivotal confirmatory stage focusing only on FL patients (*n* = 13); and (4) an expanded access fourth stage. The first three stages were reported in The New England Journal of Medicine by Pemmaraju and Lane et al. in 2019 demonstrating that among the first 29 patients enrolled and treated at the target dose of 12 µg/kg/day dosing, in the FL setting, SL-401 monotherapy yielded a 90% overall response rate including 72% rate of CR/CRc (CR or CR “clinical”–CR in all sites except minimal residual skin abnormality). They found that the median overall survival (OS) at 2 years was 52%, and 45% of patients were bridged to stem cell transplantation in the first-line treatment setting. In the R/R setting, a 67% ORR was observed among the 13 patients treated, with a median OS of 8.5 months. CLS was the most important toxicity, which was reported in approximately 20% of patients and was the cause of two deaths [1].

Based on these data, SL-401 (tagraxofusp) was granted US FDA approval on 21 December 2018, for patients with previously untreated or relapsed/refractory BPDCN ages 2 and older, and then later in the EU for adults for first-line treatment in January 2021 [20]. The CLS toxicity appropriately was designated as a “black box warning” on the package label insert [21]. This approval marked an important milestone in the field, as it was not only the first targeted agent approved specifically for patients with BPDCN, but also the first ever CD123-targeted agent approved in hematology/oncology [22,23]. Tagraxofusp is now being tested in combination with azacitidine and venetoclax in clinical trials for patients with BPDCN, AML, and myelodysplastic syndrome (MDS) with early reports of safety and efficacy reported at the American Society of Hematology Meeting in 2021 [24].

### 2.2. Monoclonal or Conjugated Antibodies

Given the high and uniform expression of CD123 in all BPDCNs, several antibody-based drugs targeting CD123 have been developed. Early efforts were composed of iterations targeting CD123 via “naked” monoclonal antibodies [25] or antibody–drug conjugates (ADCs) [25] in patients with AML, and plans to apply the same agents to BPDCN. Unfortunately, most did not reach the stage of testing in patients with BPDCN in clinical trials. However, from a disease-specific trial that is still ongoing, we now have data in patients with BPDCN using the CD123-ADC IMGN632 (ImmunoGen, Waltham, MA, USA).

IMGN632 is a humanized IgG1 monoclonal antibody specific for CD123 with a payload consisting of an indolinobenzodiazepine pseudodimer (IGN) with a peptide linker [26]. IMGN632 binds to CD123-expressing cells, is internalized, and releases FGN849, which is a potent DNA alkylating agent. Treatment of CD123+ AML cells with IMGN632 causes DNA damage, S-phase cell cycle arrest, and apoptosis [27]. IMGN632 potently kills the BPDCN cell line CAL1 and is active in vivo in patient-derived xenograft (PDX) models of BPDCN. Laboratory studies suggest that, by virtue of their lower CD123 expression, normal hematopoietic stem and progenitor cells are not as sensitive to IMGN632 as CD123+ BPDCN and AML leukemia cells [28].

IMGN632 is being tested as a single agent in patients with previously untreated or relapsed/refractory (R/R) BPDCN (ClinicalTrials.gov (accessed on 21 March 2022) Identifier: NCT03386513). Results in the first 23 patients with R/R BPDCN were presented at the American Society of Hematology meeting in December 2020 [29]. Seven of twenty-three patients had an objective response (2 CR, 2 CRc, 1 CRi, and 2 PR) for an overall response rate of 30% (95% CI, 13–53%) and a composite complete remission rate of 22%. The duration of response for the four CR/CRc patients was between 3 and 9 months, all without receiving stem cell transplantation. The drug was well tolerated with no grade 3 or higher adverse events in more than one patient. The most common grade 1–2 events were nausea, peripheral edema, and infusion-related reactions. In contrast to tagraxofusp, no capillary leak syndrome (CLS) was observed. After these data were reported, the study was expanded to include previously untreated patients with BPDCN and is ongoing in the US and Europe.

As a result of this encouraging preliminary evidence of activity, IMGN632 received breakthrough therapy designation (BTD) from the US Food and Drug Administration in October 2020 specifically for treatment of BPDCN. This grants priority review to the agent and manufacturer for future evaluation by the agency. IMGN632 is also being tested in combination with azacitidine, venetoclax, or both, in a Phase 1b/2 study for patients with AML (ClinicalTrials.gov (accessed on 21 March 2022) Identifier: NCT04086264). Based on safety and efficacy data from this study, IMGN632 combinations might also be explored in BPDCN in the future.

### 2.3. Bispecific Antibodies

Bispecific antibodies are protein drugs that have two different antigen binding sites. In general, most bispecific antibodies bind to a target on tumor cells (e.g., CD123) and a target on T cells (e.g., CD3; forming a BiTE or Bispecific T-cell Engager) to bring the immune cell in proximity to the tumor cells. This results in more specific tumor cell killing [30]. Modifications of the bispecific antibody constructs to improve activity or to recruit different immune cells, such as natural killer cells, have alternative names such as dual-affinity retargeting antibodies (DARTs) or bi-specific killer engager antibodies (BiKEs). Blinatumomab is an approved bispecific antibody for the treatment of B-cell acute lymphoblastic leukemia (B-ALL) that engages CD19 on leukemia cells and CD3 on T cells. There are no currently approved bispecific antibodies that target BPDCN surface antigens.

Results from trials using bispecific antibodies have not yet been reported in patients with BPDCN. However, encouraging results from trials testing CD123 bispecifics in AML suggest they have the potential to be active in both diseases. Flotetuzumab (MacroGenics; Rockville, MD) is an anti-CD123 x CD3 DART that has been evaluated in the setting of primary induction failure or early relapsed/refractory AML. Among 88 patients treated in an open-label phase 1/2 study, 30 patients who received the recommended phase 2 dose achieved a complete remission (CR)/CR with a partial hematologic recovery (CRh) rate of 26.7%. In patients who achieved CR/CRh, median overall survival (OS) was 10.2 months (range 1.87–27.27). A 10-gene expression signature predicted CR/CRh to flotetuzumab and correlated with bone marrow immune cell infiltration at baseline. Adverse events were mostly infusion-related reactions and cytokine release syndrome (CRS), largely grade 1–2 [31]. Capillary leak syndrome was not a prominent toxicity. Flotetuzumab is currently being tested in a basket trial for relapsed/refractory CD123-positive malignancies, including BPDCN (NCT04681105), but data from this trial have not yet been reported.

Other bispecific antibodies targeting CD123 × CD3 that are being tested in patients with AML include the BiTEs vibecotamab (XmAb14045) [32] and APVO436 [33] (that have been reported only in abstract form to date). Both have demonstrated activity in relapsed/refractory AML and have similar safety profiles as flotetuzumab, with relatively frequent but low-grade CRS. These agents may also be tested in BPDCN in the future.

### 2.4. CAR-T Cells

A CAR is a chimeric antigen receptor composed of three domains: (i) an extracellular domain that determines specificity—this is the scFv (single-chain variable fragment) of a monoclonal antibody specific to a tumor antigen; (ii) an intracellular signaling domain derived from a T-cell signaling molecule; (iii) a transmembrane domain (hinge or spacer) that links the two preceding domains and plays a role in the conformation and accessibility of the receptor to its target [34]. This strategy allows for direct recognition of tumor antigens expressed on the cell surface, independent of major histocompatibility complex presentation to T-cell receptors. Furthermore, by using the scFv of an antibody, CARs can be used to recognize a wide range of structures including proteins and non-protein structures, such as carbohydrate antigens [35].

The first CARs were developed in the late 1980s and corresponded to the variable region of a monoclonal immunoglobulin for the extracellular portion and to the regions of a TCR for the intracellular region [36,37,38,39]. However, the first “true” CAR was developed in 1993. This so-called “T-body” construct consisted of an scFv fused to the CD3ζ chain [40,41]. This first generation of CARs provided proof of concept despite limited clinical effect [42,43]. Indeed, cellular therapies modified with this type of CAR showed poor expansion and limited persistence. These weaknesses reflect a failure of T-cell activation by CARs in the absence of co-stimulatory molecules such as CD80 or CD86. The interaction with these co-stimulatory molecules is part of the three signals required for full activation of a CAR-T cell. Second- and third-generation CARs, including several co-stimulatory domains, have been developed [43,44]. These CARs that include 4-1BB or CD28 or both domains have been evaluated as a mechanism to promote tonic signaling and enhance in vivo persistence [45,46,47].

The expected promise of CARs has been highlighted with the success of anti-CD19 CARs in ALL (acute lymphocytic leukemia) and NHL (non-Hodgkin’s lymphoma), where complete remissions have been induced in numerous patients resistant to multiple lines of chemotherapy [48,49,50,51]. The first published clinical trial used a second-generation CD19-specific CAR (CD28/CD3ζ) for the treatment of ALL in relapsed adult patients [48]. In this trial, the authors evaluated 32 patients and observed a 91% response rate. Numerous clinical studies using CARs targeting CD19-positive malignancies then followed. Although each trial had its own criteria for patient recruitment and conditioning, as well as the configuration of the CAR, similar results were obtained with a response percentage between 70 and 100% in patients with leukemia and non-Hodgkin lymphoma [52,53]. Since 2017, two CD19 CARs (Tisagenlecleucel [Kymriah], Novartis; and Axicabtagene ciloleucel [Yescarta], Kite Pharma/Gilead) have been approved in the USA first, then subsequently in Europe and several other countries.

**Figure 1:**
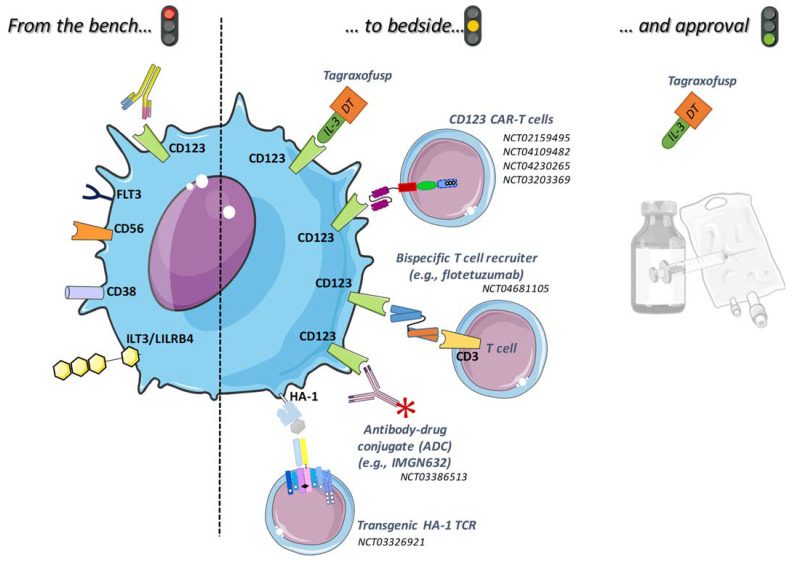
Therapies that target cell surface antigens in patients with BPDCN, highlighting tagraxofusp as the only approved therapy as well as others in clinical trials or agents and targets in earlier preclinical development.

Since 2013, several groups have developed and published preclinical studies using CD123-directed CAR-T cells [54,55,56,57,58] in AML and BPDCN (Figure 1). These CARs are often second generation, but some of them are third generation constructs based on the costimulatory and other domains used [57]. Today, 28 phase 1 or 2 CD123 CAR trials are listed in clinicaltrials.gov (accessed on 21 March 2022), mainly in AML. Most trials are open in China using second- or third-generation CARs, but none have communicated results to date.

In the USA, several CD123 CAR-T trials are ongoing, some of which include patients with BPDCN (Table 1). Cellectis conducted a clinical trial in BPDCN using allogeneic engineered T cells expressing an anti-CD123 chimeric antigen receptor. This is a second-generation CAR (CD123 scFv-41BB-CD3ζ) with a safety switch based on the RQR8 depletion ligand (confers susceptibility to the anti-CD20 monoclonal antibody rituximab). The expression of the endogenous T-cell receptor αβ (TCRαβ) is abrogated through the inactivation of the TCRα constant (TRAC) gene, using Cellectis’ TALEN gene-editing technology. In a pre-clinical study, they demonstrated a specific cytotoxic effect on BPDCN cells and prolonged survival in mouse xenograft models of BPDCN [59]. In the phase 1 trial, one patient with BPDCN was treated with a dose of 6.25 × 10^5^ CAR-T cells/kg (NCT03203369). Unfortunately, this patient had severe cytokine release syndrome (CRS) and died on day 9 after cell infusion. This trial has been closed for patients with BPDCN. However, the trial is ongoing in AML (NCT03190278) and is currently in dose escalation, with goals to evaluate the safety and clinical activity of UCART123v1.2 and to determine the maximum tolerated dose (MTD) and recommended phase 2 dose (RP2D).

Mustang Bio developed an autologous CD123 CAR-T cell product called MB-102. This CAR has a second-generation construct composed of a CD123 single-chain variable fragment, an optimized IgG4 CH2CH3 linker, with CD28 and CD3ζ signaling domains. Previously, this CAR demonstrated a strong activity on CD123+ AML cells in vitro and in vivo [54] without ablation of normal hematopoietic progenitors (CFU and BFU) from cord blood. A safety switch composed of EGFRt is incorporated in the construct, targetable by the anti-EGFR monoclonal antibody cetuximab. Today, two clinical trials are ongoing using this cell product (NCT02159495 and NCT04109482). The first one is a phase 1 trial aimed to study anti-tumor activity and safety of administration of ex vivo-expanded genetically modified T cells with two cohorts (AML and BPDCN). The second one is an expanded phase 1/2 study to assess the safety and efficacy of MB-102 in patients with relapsed or refractory BPDCN. To date, two patients with BPDCN have been treated with 100 × 10^6^ cell doses. Complete remissions have been observed with well-tolerated side-effect profiles, including only reversible and expected toxicities seen (e.g., all CRS was ≤grade 3).

Mireya Paulina Velasquez’s team in St. Jude Children’s Research Hospital is also evaluating a CD123-specific CAR in patients with recurrent/refractory CD123+ disease (AML, B-ALL, T-ALL, or BPDCN) as a bridge-to-transplant phase 1 clinical study (CATCHAML, NCT04318678). This is a second-generation CAR with a CD28 H/TM region and CD28ζ signaling domain and includes a CD20 sequence as a safety switch [60]. The University of Pennsylvania is conducting two phase 1 clinical trials using second-generation CD123 CARs based on 4.1BB and CD3ζ with different delivery methods (RNA electroporated CAR, NCT02623582; lentivirally-transduced, NCT03766126). Another trial is recruiting at the Children’s Hospital of Philadelphia (NCT04678336) to evaluate CD123 CAR-4.1BB-CD3z in pediatric AML. No results have been published to date.

In Europe, groups in France and Germany are developing CAR-T cells targeting CD123. The German group of Cartellieri et al. developed a rapidly switchable universal CAR-T platform called UniCAR that was redirected against CD123+ leukemia cells to allow a highly controlled and dose-dependent activation of the CAR-T cells [58]. This strategy is proposed to control off-target toxicities as an alternative to needing a suicide gene. This strategy is under clinical evaluation in AML, B-ALL, and BPDCN, with CD123 positivity of more than 20% of blasts is required for study entry (NCT04230265). To date, three patients with AML have been treated and no DLTs were observed. Mild and expected adverse events have been observed (grade 1 CRS, grade 1 fever) and all patients treated have shown a clinical response (two complete remissions with incomplete hematologic recovery, one partial response) [61].

In France, Garnache-Ottou and team investigated the anti-leukemia efficacy and safety of a third generation lentiviral CD28/4-1BB CAR-T cell product targeting CD123 (CAR123), and provided strong preclinical rationale for the clinical assessment of this autologous cell therapy [57]. This CAR is currently undergoing clinical translation to meet good manufacturing practice (GMP) requirements using a closed automated system. A phase 1/2 clinical trial to validate the clinical proof of concept of CAR123 for patients with BPDCN will be the next step and is planned.

### 2.5. Promising New Targets

#### 2.5.1. CD38

CD38 is a cell surface antigen that is expressed or overexpressed in several different hematologic malignancies including multiple myeloma (MM), T-cell acute lymphoblastic leukemia (T-ALL), and others including some BPDCNs. In BPDCN, CD38 is not usually included as part of the standard diagnostic workup, but of interest, several groups have demonstrated that it can be expressed in BPDCN, with perhaps 50% or more of cases being positive [62]. This finding is not only of potential diagnostic relevance, but also may be considered therapeutically important. The monoclonal antibody agent known as daratumumab is an already available, approved targeted therapy directed against CD38, currently used in patients with MM. Iverson et al. demonstrated monotherapy activity with daratumumab in a 70-year-old patient with untreated CD38+ BPDCN [63]. Another recent report by Mirgh et al. demonstrated efficacy of daratumumab in combination with bortezomib in a 75-year-old patient with BPDCN, who had extensive CD38+ disease involvement that was relapsed/refractory after standard treatments [64]. Therefore, it would be of interest for the BPDCN field to perform further studies both into expression of CD38+ in BPDCN and development of formal clinical trials for monotherapy and combination studies with daratumumab or other novel immunotherapies in CD38+ BPDCN.

#### 2.5.2. HA-1H

CARs directed at cell-surface targets associated with other more common tumor types may also be adapted for treating BPDCN. For example, the minor histocompatibility antigen HA-1 is exclusively expressed on hematopoietic cells and is presented to the immune system in the context of HLA-A*02:01 [65]. The antigenic HA-1H isoform is relatively common in the general population and TCR gene transfer can create HA-1H antigen-specific T cells, including in the setting of allogeneic stem cell transplantation [66]. Therefore, a basket CAR-T cell trial is underway for patients with multiple types of leukemias, including BPDCN, that express HLA-A*02:01 and who have persistent or relapsed disease after an allogeneic SCT from an HLA-A*02:01 or HA-1H negative donor (clinicaltrials.gov (accessed on 21 March 2022): NCT03326921). No data have been released to date.

#### 2.5.3. CD56

As CD56 is also expressed on BPDCN blasts, and this antigen could be a target to eliminate leukemia cells. Some strategies using antibody or CAR-T cells targeting CD56 are under evaluation in other pathologies, suggesting these strategies could also be evaluated in BPDCN. For example, a new CD56-targeting monomethyl auristatin E-conjugated antibody–drug conjugate is active in preclinical models of Merkel cell carcinoma [67]. Similarly, a CD56-targeted CAR-T is active in models of small-cell lung cancer and neuroblastoma [68]. These preclinical data indicate that CD56-targeted therapies merit further investigation as a potential treatment for CD56+ hematologic malignancies such as BPDCN. However, CD56 is also expressed on NK cells and a subset of T cells. Therefore, CD56-directed therapy, particularly if long-lasting such as via anti-CD56 CAR-T cells, may need to be used as a bridge to hematopoietic stem cell transplantation, which would eliminate residual CAR-T cells and restore CD56+ lymphocytes.

#### 2.5.4. ILT3

Immunoglobulin-like transcript 3, ILT3, encoded by the gene LILRB4, is an important cell surface regulator of dendritic cell function [69]. ILT3 is highly expressed on normal pDCs and monocytes, and similarly is expressed on most BPDCNs and monocytic (FAB subtype M4/M5) AMLs. An ILT3/LILRB4-directed CAR-T cell preclinical model was active against monocytic AML cells and was not toxic to normal progenitors derived from normal CD34+ umbilical cord blood in vitro or humanized hematopoiesis in a mouse model [70]. These data suggest that an ILT3-directed immunotherapy could also be tested in patients with BPDCN.

## 3. Conclusions

Despite the significant increase in disease-specific research on BPDCN in recent years, patients are still in need of better treatments. The success of tagraxofusp demonstrated the potential for targeting CD123 and for the discovery and evaluation of BPDCN-specific therapies as a feasible drug-development endeavor. This strategy may be particularly effective in the long-term when there are target antigens, like CD123, that are shared with other cancers. This can expand the reach of novel agents to more patients while also directly helping those with BPDCN. Several other classes of immunotherapeutic agents that also target CD123 are in development for other hematologic malignancies, as outlined here. We hope that design of preclinical experiments and clinical trials will include BPDCN where possible, to extend the toolbox of therapies in this still orphan disease. We also propose that there are even advantages to making the initial focus of an agent’s development on an orphan disease indication, such as BPDCN, as “proof-of-concept.” This is particularly helpful if the orphan disease is relatively homogenous between patients and if the target is highly expressed and/or essential for the biology of the malignant cell. Dramatic responses only require small patient numbers to support early clinical evaluation. For these reasons, we believe that several additional targets and therapies being tested in other malignancies also hold promise for co-development in BPDCN. A thoughtful approach in this way is essential to bring novel therapies to rare diseases that would otherwise be less likely to be selected for drug development on their own.

## Figures and Tables

**Table 1:** Clinical trials using CAR-T cells targeting CD123 in patients with leukemia including BPDCN and active non-CAR-T cell BPDCN trials (extracted from clinicaltrials.gov (accessed on 21 March 2022)).

CD123 CAR T-Cell Trials						
NCT	System	Safety Switch	Condition/Disease	Dose	Phase	Status
**NCT04318678**	CD123-CAR CD28 TM-CD28-CD3z	CD20	AML, B-ALL, T-ALL, BPDCN	3 × 10^5^, 1 × 10^6^, 3 × 10^6^, 1 × 10^7^ cells/kg	1	Recruiting
**NCT02159495**	CD123-CAR IgG4 TM-CD28-CD3z	EGFRt	CD123+ diseases		1	Recruiting
**NCT04109482**	CD123-CAR IgG4 TM-CD28-CD3z (MB-102)	EGFRt	BPDCN	Up to 600 × 10^6^ cells	1/2	Recruiting
**NCT02623582**	CD123-CAR 41BB-CD3z (RNA electroporated)		AML		Early phase 1	Terminated
**NCT03766126**	CD123-CAR 41BB-CD3z (lentiviral transduced)		AML	1–5 × 10^6^ cells/kg	1	Active, not recruiting
**NCT03203369**	Allogenic UCART123-41BB-CD3z	RQR8	BPDCN	6.25 × 10^5^ –6.25 × 10^6^ cells/kg	1	Terminated
**NCT03190278**	Allogenic UCART123 v1.2 -41BB-CD3z	RQR8	AML		1	Recruiting
**NCT04678336**	CD123-CAR 41BB-CD3z		Pediatric AML	2 × 10^6^ cells/kg	1	Recruiting
**Other active non-CAR-T cell BPDCN trials**						
**NCT**	Agent(s)		Condition/Disease		Phase	Status
**NCT03113643**	SL-401, venetoclax, azacitidine		BPDCN, AML, MDS		1	Recruiting
**NCT03386513**	IMGN632		BPDCN		1/2	Recruiting
**NCT04216524**	SL-401, venetoclax, Hyper-CVAD		BPDCN		1	Recruiting
**NCT04317781**	SL-401		BPDCN after stem cell transplant		2	Active, not recruiting

## References

[1] Pemmaraju N., Lane A.A., Sweet K.L., Stein A.S., Vasu S., Blum W., Rizzieri D.A., Wang E.S., Duvic M., Sloan J.M. (2019). Tagraxofusp in Blastic Plasmacytoid Dendritic-Cell Neoplasm. N. Engl. J. Med..

[2] Montero J., Stephansky J., Cai T., Griffin G.K., Cabal-Hierro L., Togami K., Hogdal L.J., Galinsky I., Morgan E.A., Aster J.C. (2017). Blastic Plasmacytoid Dendritic Cell Neoplasm Is Dependent on BCL2 and Sensitive to Venetoclax. Cancer Discov..

[3] Philippe L., Ceroi A., Bôle-Richard E., Jenvrin A., Biichle S., Perrin S., Limat S., Bonnefoy F., Deconinck E., Saas P. (2017). Bortezomib as a New Therapeutic Approach for Blastic Plasmacytoid Dendritic Cell Neoplasm. Haematologica.

[4] Marmouset V., Joris M., Merlusca L., Beaumont M., Charbonnier A., Marolleau J.-P., Gruson B. (2019). The Lenalidomide/Bortezomib/Dexamethasone Regimen for the Treatment of Blastic Plasmacytoid Dendritic Cell Neoplasm. Hematol. Oncol..

[5] Sapienza M.R., Fuligni F., Agostinelli C., Tripodo C., Righi S., Laginestra M.A., Pileri A., Mancini M., Rossi M., Ricci F. (2014). Molecular Profiling of Blastic Plasmacytoid Dendritic Cell Neoplasm Reveals a Unique Pattern and Suggests Selective Sensitivity to NF-KB Pathway Inhibition. Leukemia.

[6] Garnache-Ottou F., Vidal C., Biichlé S., Renosi F., Poret E., Pagadoy M., Desmarets M., Roggy A., Seilles E., Soret L. (2019). How Should We Diagnose and Treat Blastic Plasmacytoid Dendritic Cell Neoplasm Patients?. Blood Adv..

[7] Dalle S., Beylot-Barry M., Bagot M., Lipsker D., Machet L., Joly P., Dompmartin A., D’Incan M., Maubec E., Grange F. (2010). Blastic Plasmacytoid Dendritic Cell Neoplasm: Is Transplantation the Treatment of Choice?. Br. J. Dermatol..

[8] Pagano L., Valentini C.G., Grammatico S., Pulsoni A. (2016). Blastic Plasmacytoid Dendritic Cell Neoplasm: Diagnostic Criteria and Therapeutical Approaches. Br. J. Haematol..

[9] Roos-Weil D., Dietrich S., Boumendil A., Polge E., Bron D., Carreras E., Iriondo Atienza A., Arcese W., Beelen D.W., Cornelissen J.J. (2013). Stem Cell Transplantation Can Provide Durable Disease Control in Blastic Plasmacytoid Dendritic Cell Neoplasm: A Retrospective Study from the European Group for Blood and Marrow Transplantation. Blood.

[10] Bashir Q., Milton D.R., Popat U.R., Kebriaei P., Hosing C., Khouri I.F., Rezvani K., Nieto Y., Oran B., Srour S.A. (2021). Allogeneic Hematopoietic Cell Transplantation for Patients with Blastic Plasmacytoid Dendritic Cell Neoplasm (BPDCN). Bone Marrow Transpl..

[11] Schreiber R.D., Old L.J., Smyth M.J. (2011). Cancer Immunoediting: Integrating Immunity’s Roles in Cancer Suppression and Promotion. Science.

[12] Couzin-Frankel J. (2013). Cancer Immunotherapy. Science.

[13] Bridgeman J.S., Hawkins R.E., Bagley S., Blaylock M., Holland M., Gilham D.E. (2010). The Optimal Antigen Response of Chimeric Antigen Receptors Harboring the CD3ζ Transmembrane Domain Is Dependent upon Incorporation of the Receptor into the Endogenous TCR/CD3 Complex. J. Immunol..

[14] Mezzanzanica D., Canevari S., Mazzoni A., Figini M., Colnaghi M.I., Waks T., Schindler D.G., Eshhar Z. (1998). Transfer of Chimeric Receptor Gene Made of Variable Regions of Tumor-Specific Antibody Confers Anticarbohydrate Specificity on T Cells. Cancer Gene Ther..

[15] Becker M.L.B., Near R., Mudgett-Hunter M., Margolies M.N., Kubo R.T., Kaye J., Hedrick S.M. (1989). Expression of a Hybrid Immunoglobulin-T Cell Receptor Protein in Transgenic Mice. Cell.

[16] Goverman J., Gomez S.M., Segesman K.D., Hunkapiller T., Laug W.E., Hood L. (1990). Chimeric Immunoglobulin-T Cell Receptor Proteins Form Functional Receptors: Implications for T Cell Receptor Complex Formation and Activation. Cell.

[17] Gross G., Waks T., Eshhar Z. (1989). Expression of Immunoglobulin-T-Cell Receptor Chimeric Molecules as Functional Receptors with Antibody-Type Specificity. Proc. Natl. Acad. Sci. USA.

[18] Kuwana Y., Asakura Y., Utsunomiya N., Nakanishi M., Arata Y., Itoh S., Nagase F., Kurosawa Y. (1987). Expression of Chimeric Receptor Composed of Immunoglobulin-Derived V Regions and T-Cell Receptor-Derived C Regions. Biochem. Biophys. Res. Commun..

[19] Eshhar Z., Waks T., Gross G., Schindler D.G. (1993). Specific Activation and Targeting of Cytotoxic Lymphocytes through Chimeric Single Chains Consisting of Antibody-Binding Domains and the Gamma or Zeta Subunits of the Immunoglobulin and T-Cell Receptors. Proc. Natl. Acad. Sci. USA.

[20] Eshhar Z., Bach N., Fitzer-Attas C.J., Grosse G., Lustgarten J., Waks T., Schindler D.G. (1996). The T-Body Approach: Potential for Cancer Immunotherapy. Springer Semin. Immunopathol..

[21] Hwu P., Yang J.C., Cowherd R., Treisman J., Shafer G.E., Eshhar Z., Rosenberg S.A. (1995). In Vivo Antitumor Activity of T Cells Redirected with Chimeric Antibody/T-Cell Receptor Genes. Cancer Res..

[22] Ramos C.A., Dotti G. (2011). Chimeric Antigen Receptor (CAR)-Engineered Lymphocytes for Cancer Therapy. Expert Opin. Biol. Ther..

[23] Sadelain M., Brentjens R., Riviere I. (2013). The Basic Principles of Chimeric Antigen Receptor (CAR) Design. Cancer Discov..

[24] Wang J., Jensen M., Lin Y., Sui X., Chen E., Lindgren C.G., Till B., Raubitschek A., Forman S.J., Qian X. (2007). Optimizing Adoptive Polyclonal T Cell Immunotherapy of Lymphomas, Using a Chimeric T Cell Receptor Possessing CD28 and CD137 Costimulatory Domains. Hum. Gene Ther..

[25] Stoiber S., Cadilha B.L., Benmebarek M.-R., Lesch S., Endres S., Kobold S. (2019). Limitations in the Design of Chimeric Antigen Receptors for Cancer Therapy. Cells.

[26] Gomes-Silva D., Mukherjee M., Srinivasan M., Krenciute G., Dakhova O., Zheng Y., Cabral J.M.S., Rooney C.M., Orange J.S., Brenner M.K. (2017). Tonic 4-1BB Costimulation in Chimeric Antigen Receptors Impedes T Cell Survival and Is Vector Dependent. Cell Rep..

[27] Angelot-Delettre F., Roggy A., Frankel A.E., Lamarthee B., Seilles E., Biichle S., Royer B., Deconinck E., Rowinsky E.K., Brooks C. (2015). In Vivo and in Vitro Sensitivity of Blastic Plasmacytoid Dendritic Cell Neoplasm to SL-401, an Interleukin-3 Receptor Targeted Biologic Agent. Haematologica.

[28] Garnache-Ottou F., Feuillard J., Ferrand C., Biichle S., Trimoreau F., Seilles E., Salaun V., Garand R., Lepelley P., Maynadié M. (2009). Extended Diagnostic Criteria for Plasmacytoid Dendritic Cell Leukaemia. Br. J. Haematol..

[29] Testa U., Pelosi E., Frankel A. (2014). CD 123 Is a Membrane Biomarker and a Therapeutic Target in Hematologic Malignancies. Biomark Res..

[30] Frankel A., Liu J.-S., Rizzieri D., Hogge D. (2008). Phase I Clinical Study of Diphtheria Toxin-Interleukin 3 Fusion Protein in Patients with Acute Myeloid Leukemia and Myelodysplasia. Leuk. Lymphoma.

[31] FitzGerald D.J. (2014). Targeted Diphtheria Toxin to Treat BPDCN. Blood.

[32] Frankel A.E., Woo J.H., Ahn C., Pemmaraju N., Medeiros B.C., Carraway H.E., Frankfurt O., Forman S.J., Yang X.A., Konopleva M. (2014). Activity of SL-401, a Targeted Therapy Directed to Interleukin-3 Receptor, in Blastic Plasmacytoid Dendritic Cell Neoplasm Patients. Blood.

[33] Pemmaraju N., Konopleva M. (2020). Approval of Tagraxofusp-Erzs for Blastic Plasmacytoid Dendritic Cell Neoplasm. Blood Adv..

[34] Wilson N.R., Konopleva M., Khoury J.D., Pemmaraju N. (2021). Novel Therapeutic Approaches in Blastic Plasmacytoid Dendritic Cell Neoplasm (BPDCN): Era of Targeted Therapy. Clin. Lymphoma Myeloma Leuk..

[35] Patnaik M.M., Mughal T.I., Brooks C., Lindsay R., Pemmaraju N. (2021). Targeting CD123 in Hematologic Malignancies: Identifying Suitable Patients for Targeted Therapy. Leuk. Lymphoma.

[36] Lane A.A. (2020). Targeting CD123 in AML. Clin. Lymphoma Myeloma Leuk..

[37] Lane A.A., Stein A.S., Garcia J.S., Garzon J.L., Galinsky I., Luskin M.R., Stone R.M., Winer E.S., Leonard R., Mughal T.I. (2021). Safety and Efficacy of Combining Tagraxofusp (SL-401) with Azacitidine or Azacitidine and Venetoclax in a Phase 1b Study for CD123 Positive AML, MDS, or BPDCN. Blood.

[38] He S.Z., Busfield S., Ritchie D.S., Hertzberg M.S., Durrant S., Lewis I.D., Marlton P., McLachlan A.J., Kerridge I., Bradstock K.F. (2015). A Phase 1 Study of the Safety, Pharmacokinetics and Anti-Leukemic Activity of the Anti-CD123 Monoclonal Antibody CSL360 in Relapsed, Refractory or High-Risk Acute Myeloid Leukemia. Leuk. Lymphoma.

[39] Li F., Sutherland M.K., Yu C., Walter R.B., Westendorf L., Valliere-Douglass J., Pan L., Cronkite A., Sussman D., Klussman K. (2018). Characterization of SGN-CD123A, A Potent CD123-Directed Antibody–Drug Conjugate for Acute Myeloid Leukemia. Mol. Cancer Ther..

[40] Archer K.E., Reid E.E., Shizuka M., Woods J., Harris L., Maloney E.K., Bartle L.M., Ab O., Wilhelm A., Setiady Y. (2019). Synthesis of Highly Potent N-10 Amino-Linked DNA-Alkylating Indolinobenzodiazepine Antibody–Drug Conjugates (ADCs). ACS Med. Chem. Lett..

[41] Kovtun Y., Noordhuis P., Whiteman K.R., Watkins K., Jones G.E., Harvey L., Lai K.C., Portwood S., Adams S., Sloss C.M. (2018). IMGN779, a Novel CD33-Targeting Antibody–Drug Conjugate with DNA-Alkylating Activity, Exhibits Potent Antitumor Activity in Models of AML. Mol. Cancer Ther..

[42] Kovtun Y., Jones G.E., Adams S., Harvey L., Audette C.A., Wilhelm A., Bai C., Rui L., Laleau R., Liu F. (2018). A CD123-Targeting Antibody-Drug Conjugate, IMGN632, Designed to Eradicate AML While Sparing Normal Bone Marrow Cells. Blood Adv..

[43] Pemmaraju N., Martinelli G., Todisco E., Lane A.A., Acuña-Cruz E., Deconinck E., Wang E.S., Sweet K.L., Rizzieri D.A., Mazzarella L. (2020). Clinical Profile of IMGN632, a Novel CD123-Targeting Antibody-Drug Conjugate (ADC), in Patients with Relapsed/Refractory (R/R) Blastic Plasmacytoid Dendritic Cell Neoplasm (BPDCN). Blood.

[44] Allen C., Zeidan A.M., Bewersdorf J.P. (2021). BiTEs, DARTS, BiKEs and TriKEs—Are Antibody Based Therapies Changing the Future Treatment of AML?. Life.

[45] Uy G.L., Aldoss I., Foster M.C., Sayre P.H., Wieduwilt M.J., Advani A.S., Godwin J.E., Arellano M.L., Sweet K.L., Emadi A. (2021). Flotetuzumab as Salvage Immunotherapy for Refractory Acute Myeloid Leukemia. Blood.

[46] Ravandi F., Bashey A., Foran J.M., Stock W., Mawad R., Blum W., Saville M.W., Johnson C.M., Vanasse K.G.J., Ly T. (2018). Complete Responses in Relapsed/Refractory Acute Myeloid Leukemia (AML) Patients on a Weekly Dosing Schedule of XmAb14045, a CD123 x CD3 T Cell-Engaging Bispecific Antibody: Initial Results of a Phase 1 Study. Blood.

[47] Watts J.M., Lin T., Wang E.S., Mims A.S., Cull E.H., Patel P.A., Shami P.J., Walter R.B., Cogle C.R., Chenault R.A. (2020). Preliminary Results from a Phase 1 Study of APVO436, a Novel Anti-CD123 x Anti-CD3 Bispecific Molecule, in Relapsed/Refractory Acute Myeloid Leukemia and Myelodysplastic Syndrome. Blood.

[48] Brentjens R., Davila M.L., Riviere I., Park J., Wang X., Cowell L.G., Bartido S., Stefanski J., Taylor C., Olszewska M. (2013). CD19-Targeted T Cells Rapidly Induce Molecular Remissions in Adults with Chemotherapy-Refractory Acute Lymphoblastic Leukemia. Sci. Transl. Med..

[49] Davila M.L., Riviere I., Wang X., Bartido S., Park J., Curran K., Chung S.S., Stefanski J., Borquez-Ojeda O., Olszewska M. (2014). Efficacy and Toxicity Management of 19-28z CAR T Cell Therapy in B Cell Acute Lymphoblastic Leukemia. Sci. Transl. Med..

[50] Grupp S.A., Kalos M., Barrett D., Aplenc R., Porter D.L., Rheingold S.R., Teachey D.T., Chew A., Hauck B., Wright J.F. (2013). Chimeric Antigen Receptor–Modified T Cells for Acute Lymphoid Leukemia. New Engl. J. Med..

[51] Kochenderfer J.N., Dudley M.E., Kassim S.H., Somerville R.P.T., Carpenter R.O., Stetler-Stevenson M., Yang J.C., Phan G.Q., Hughes M.S., Sherry R.M. (2015). Chemotherapy-Refractory Diffuse Large B-Cell Lymphoma and Indolent B-Cell Malignancies Can Be Effectively Treated With Autologous T Cells Expressing an Anti-CD19 Chimeric Antigen Receptor. J. Clin. Oncol..

[52] Sadelain M. (2015). CAR Therapy: The CD19 Paradigm. J. Clin. Investig..

[53] Maude S.L., Laetsch T.W., Buechner J., Rives S., Boyer M., Bittencourt H., Bader P., Verneris M.R., Stefanski H.E., Myers G.D. (2018). Tisagenlecleucel in Children and Young Adults with B-Cell Lymphoblastic Leukemia. N. Engl. J. Med..

[54] Mardiros A., Dos Santos C., McDonald T., Brown C.E., Wang X., Budde L.E., Hoffman L., Aguilar B., Chang W.-C., Bretzlaff W. (2013). T Cells Expressing CD123-Specific Chimeric Antigen Receptors Exhibit Specific Cytolytic Effector Functions and Antitumor Effects against Human Acute Myeloid Leukemia. Blood.

[55] Gill S., Tasian S.K., Ruella M., Shestova O., Li Y., Porter D.L., Carroll M., Danet-Desnoyers G., Scholler J., Grupp S.A. (2014). Preclinical Targeting of Human Acute Myeloid Leukemia and Myeloablation Using Chimeric Antigen Receptor–Modified T Cells. Blood.

[56] Tasian S.K., Kenderian S.S., Shen F., Ruella M., Shestova O., Kozlowski M., Li Y., Schrank-Hacker A., Morrissette J.J.D., Carroll M. (2017). Optimized Depletion of Chimeric Antigen Receptor T Cells in Murine Xenograft Models of Human Acute Myeloid Leukemia. Blood.

[57] Bôle-Richard E., Fredon M., Biichlé S., Anna F., Certoux J.-M., Renosi F., Tsé F., Molimard C., Valmary-Degano S., Jenvrin A. (2020). CD28/4-1BB CD123 CAR T Cells in Blastic Plasmacytoid Dendritic Cell Neoplasm. Leukemia.

[58] Loff S., Dietrich J., Meyer J.-E., Riewaldt J., Spehr J., von Bonin M., Gründer C., Swayampakula M., Franke K., Feldmann A. (2020). Rapidly Switchable Universal CAR-T Cells for Treatment of CD123-Positive Leukemia. Mol. Ther. Oncolytics..

[59] Cai T., Galetto R., Gouble A., Smith J., Cavazos A., Konoplev S., Lane A.A., Guzman M.L., Kantarjian H.M., Pemmaraju N. (2016). Pre-Clinical Studies of Anti-CD123 CAR-T Cells for the Treatment of Blastic Plasmacytoid Dendritic Cell Neoplasm (BPDCN). Blood.

[60] Riberdy J.M., Zhou S., Zheng F., Kim Y.-I., Moore J., Vaidya A., Throm R.E., Sykes A., Sahr N., Bonifant C.L. (2020). The Art and Science of Selecting a CD123-Specific Chimeric Antigen Receptor for Clinical Testing. Mol. Ther.—Methods Clin. Dev..

[61] Wermke M., Kraus S., Ehninger A., Bargou R.C., Goebeler M.-E., Middeke J.M., Kreissig C., von Bonin M., Koedam J., Pehl M. (2021). Proof of Concept for a Rapidly Switchable Universal CAR-T Platform with UniCAR-T-CD123 in Relapsed/Refractory AML. Blood.

[62] Deotare U., Yee K.W.L., Le L.W., Porwit A., Tierens A., Musani R., Barth D., Torlakovic E., Schimmer A., Schuh A.C. (2016). Blastic Plasmacytoid Dendritic Cell Neoplasm with Leukemic Presentation: 10-Color Flow Cytometry Diagnosis and HyperCVAD Therapy. Am. J. Hematol..

[63] Iversen K.F., Holdgaard P.C., Preiss B., Nyvold C.G., Plesner T. (2019). Daratumumab for Treatment of Blastic Plasmacytoid Dendritic Cell Neoplasm. A Single-Case Report. Haematologica.

[64] Mirgh S., Sharma A., Folbs B., Khushoo V., Kapoor J., Tejwani N., Ahmed R., Agrawal N., Choudhary P.S., Mehta P. (2021). Daratumumab-Based Therapy after Prior Azacytidine-Venetoclax in an Octagenerian Female with BPDCN (Blastic Plasmacytoid Dendritic Cell Neoplasm)—A New Perspective. Leuk. Lymphoma.

[65] van Loenen M.M., de Boer R., Hagedoorn R.S., van Egmond E.H.M., Falkenburg J.H.F., Heemskerk M.H.M. (2011). Optimization of the HA-1-Specific T-Cell Receptor for Gene Therapy of Hematologic Malignancies. Haematologica.

[66] van Balen P., Jedema I., van Loenen M.M., de Boer R., van Egmond H.M., Hagedoorn R.S., Hoogstaten C., Veld S.A.J., Hageman L., van Liempt P.A.G. (2020). HA-1H T-Cell Receptor Gene Transfer to Redirect Virus-Specific T Cells for Treatment of Hematological Malignancies After Allogeneic Stem Cell Transplantation: A Phase 1 Clinical Study. Front. Immunol..

[67] Esnault C., Leblond V., Martin C., Desgranges A., Baltus C.B., Aubrey N., Lakhrif Z., Lajoie L., Lantier L., Clémenceau B. (2021). Adcitmer^®^, a New CD56-Targeting Monomethyl Auristatin E-Conjugated Antibody, Is a Potential Therapeutic Approach in Merkel Cell Carcinoma. Br. J. Dermatol..

[68] Crossland D.L., Denning W.L., Ang S., Olivares S., Mi T., Switzer K., Singh H., Huls H., Gold K.S., Glisson B.S. (2018). Antitumor Activity of CD56-Chimeric Antigen Receptor T Cells in Neuroblastoma and SCLC Models. Oncogene.

[69] Vlad G., Chang C.-C., Colovai A.I., Berloco P., Cortesini R., Suciu-Foca N. (2009). Immunoglobulin-like Transcript 3: A Crucial Regulator of Dendritic Cell Function. Hum. Immunol..

[70] John S., Chen H., Deng M., Gui X., Wu G., Chen W., Li Z., Zhang N., An Z., Zhang C.C. (2018). A Novel Anti-LILRB4 CAR-T Cell for the Treatment of Monocytic AML. Mol. Ther..

